# Supplementary material for: Are carbon emissions trading and green financial instruments synergistic? -Comprehensive quantitative research based on content analysis
Source: PLoS One. 2024 Mar 7;19(3):e0298601. doi: 10.1371/journal.pone.0298601 (PMC10919601; doi:10.1371/journal.pone.0298601)
Supplement: S1 Data — (ZIP) [file pone.0298601.s001.zip › Data/地级市Z_F工作报告.zip!/╜¡╬≈/╛░╡┬╒≥╩╨/└┤╫╘╣1⁄2╓┌║┼í╛┬φ┐╦╩2╛▌═°í┐_2009.docx]

各位代表：

现在，我代表市人民政府向大会作政府工作报告，请予审议，并请各位市政协委员和列席会议的同志提出意见。

一、2008年的工作回顾

过去的一年，在省委、省政府和市委的正确领导下，全市上下坚持以邓小平理论和“三个代表”重要思想为指导，全面落实党的十七大和十七届三中全会精神，紧紧围绕市委九届四次全会提出的“三个40%、力争50%，确保实现两年翻番”的发展目标，突出增加投入、做大总量，克服了低温雨雪冰冻灾害、较大洪灾和国际金融危机等不利影响，全市经济社会实现了快速、健康、协调发展。

2008年，全市实现生产总值321.98亿元，增长15.3%；社会消费品零售总额99.28亿元，增长24.1%；城镇居民人均可支配收入13591元，增长16.8%；农民人均纯收入5253元，增长17.45%；人口自然增长率7.88‰。

三大主要指标快速增长。完成财政总收入28.4亿元，增长41.8%；完成地方财政收入18.5亿元，增长55.6%，两项指标增幅均列全省第一。四个县（市、区）财政全部实现“三年翻番”。完成城镇以上固定资产投资222亿元，增长105.7%，为建国以来我市最高水平，净增113.96亿元，增量超过2007年全年的总和，我市被评为全省三个固定资产投资先进设区市之一。规模以上工业企业新增83家，完成规模以上工业增加值131.25亿元，增长22.2%。陶瓷产业实现新跨越，全市陶瓷工业总产值完成70亿元，增长70%；陶瓷出口7050万美元，增长40%；陶瓷产业税收8399万元，增长94%。

项目建设取得重大突破。坚持大开放主战略，实际引进内资96.8亿元，增长100.9%；实际引进外资8765万美元，增长12.2%；出口完成2.2亿美元，增长33.9%。产业项目加快推进，投资50亿元的光伏产业项目、投资35亿元的陶瓷产业项目、投资50亿元的汽车机电项目、投资14亿元的长虹家电项目、投资5亿元的LED照明等重大产业项目相继落地、开工，进展顺利。一批重大基础设施项目的争取工作实现历史性突破，投资8亿元的天然气项目、投资1.4亿元的机场扩建工程正式开工建设；国家能源局批准同意投资50亿元的电厂“上大压小”项目开展前期工作；在我市境内投资59亿元的九景衢铁路和皖赣铁路复线改造工程确定今年开工。2008瓷博会成功举办，内贸成交总额8.06亿元，外贸成交总额1.54亿美元。

农村经济稳步发展。实现农业总产值48亿元，增长5.6%。粮食总产再创历史新高，达到54.5万吨，增长5.8%。农业农村基础设施建设得到加强，“造地增粮富民”工程加快推进，完成造地7100亩。造林绿化“一大四小”工程开局良好，完成造林面积12.9万亩。凝聚着几届政府努力和全市人民愿望、投资约22.16亿的浯溪口水利枢纽工程项目建议书获得国家发改委正式批复。社会主义新农村建设稳步推进，累计完成投入1.32亿元。

城市面貌明显改善。以创建全国文明城市为抓手，城市功能不断完善，再次获得全国创建文明城市工作先进城市称号。正式启动新一轮城市总体规划修编工作，城区详规覆盖率达88.2%。重点工程建设加快，白鹭大桥胜利竣工；湖田大桥建成通车；西瓜洲城市污水处理厂投入运营，结束了我市城市污水直接排放昌江河的历史；体育中心完成一期主体育场土建工程；景德镇陶瓷艺术研究院基本完工；中山路、中华路、沿江东路全线改造完成并通车，沿江西路改造工程基本完成；改造里弄214条；莲花塘环山休闲小道修建完工；城市森林公园、景德镇民窑博物馆、珠山八友广场、景德镇国动委大楼等工程开工建设；完成曙光路、昌江大桥东头、加州阳光路口等5个文艺小广场建设，广场立交桥、6个景观戏亭建设即将完工。在全省率先推行城市数字化、网格化管理。顺利完成出租车更新换型。严厉打击了违法用地和违章建筑。矿产资源开发秩序得到规范。绿色生态瓷都建设取得成效，城市全年空气质量优良率达100%。土地运作逆市增长，经营性土地出让成交量排在全省前列，土地出让净收益6.28亿元，出让均价增长24%；利用土地融资5.45亿元，市本级直接拨付高新区和陶瓷工业园区建设资金3.3亿元；报省政府批准建设用地指标4100亩，为园区提供工业用地3200亩，对园区投入和土地供应均为我市历史以来最多的一年。同时，通过土地运作支付政府性贷款利息2.25亿元，消除银行不良贷款1.4亿元，收储闲置经营性土地1800亩，有效地补充了财力和各项建设资金。

旅游产业健康发展。全年接待国内外游客908万人次，增长18.3%，实现旅游总收入44亿元，增长45.4%。景区景点建设加快，陶瓷历史文化博览区、御窑厂、高岭-瑶里风景区、浮梁古县衙等重点景区功能进一步完善。得雨生态园和洪岩风景区成功创建国家AAAA级景区。浮梁县成功创建江西旅游强县。瑶里镇、瑶里村分别被评为全国环境优美乡镇和国家级生态村。旅游市场不断拓展，先后组织参加了中国国际及国内旅游交易会、红色旅游产品交易会等一系列旅游推介会。《大瓷商》开拍、组织邀请中央电视台等新闻媒体和名人访谈等活动，进一步扩大了我市陶瓷文化旅游的影响力。

各项改革深入推进。国有企业改革稳步实施，完成28户企业的改革改制工作，全市国有企业改革改制面达72.6%。焦化总厂公司制改造和华意压缩战略重组工作完成。省属12户国有企业顺利交接。国有资产监管和运营不断加强，受理了29户企业产权交易业务，完成第一批机关事业单位国有资产统一划转工作，收购不良资产包20亿元。财政改革扎实推进，非税收入管理进一步规范，市直50个单位国库集中支付改革按时完成，房地产税费实行了“一体化”征管。农村综合改革积极推进，林业产权体制主体改革基本完成，配套改革全面推进；种子管理体制、水利管理体制、基层服务体系等改革扎实开展。

民生工程扎实有效。城镇新增就业3.07万人，城镇就业率96.5%，新增转移农村劳动力3.7万人。城镇职工和农民工参加基本养老保险分别达到32.5万人和2.56万人，农民参加新型合作医疗达到86.41万人。低保标准进一步提高，城市和农村低保补差水平分别达到141.9元和64.8元，农村五保户集中供养率达到83%。开工建设经济适用住房10万平方米，受益家庭1670户，新建廉租房108套，廉租保障家庭1397户。新增解决农村安全饮水2.55万人，农村使用沼气池新增3102户。完成农村公路改造273公里。

和谐平安创建成效显著。深入开展严打整治斗争，严厉打击了严重刑事犯罪活动。社会治安综合治理深入推进，治安防控体系不断健全，社会治安好转率列全省第一，公众安全感满意率不断提高。“五五”普法和依法治理工作深入推进。安全生产监管进一步加强，非法小煤窑继续实现了“零开采、零死亡”，全市安全生产连续七年未发生一起死亡10人以上的重特大事故。维稳工作机制不断健全，信访维稳态势总体良好，圆满完成了奥运会等重大活动时期赴京非正常上访为零的目标。

社会事业全面进步。国家日用及建筑陶瓷工程中心通过科技部验收，并被批准为首批国家技术转移示范机构。国家陶瓷火炬计划基地落户我市。与中科院开展科技合作取得突破性进展。科技创新取得新成效,申报国家和省科技项目73项，立项42项。陶瓷工程中心项目获国家科技进步二等奖。全年专利申请量206件。公民平均受教育年限在全省领先。全面实施城乡免费义务教育，新五中建设工程快速推进，梨树园小学开工新建。优质高中工程全面完成，高考录取率高于全省平均水平5.8个百分点。职业教育基础能力建设力度加大。老年教育进一步发展。海畅公司成功申报全国文化产业示范基地。非物质文化遗产保护取得实效，“景德镇传统青花瓷制作技艺”等6项非物质文化遗产入选省级保护名录。10名老技工、老艺人纳入第一批传统制瓷代表性传承人管理与保护。我市获得“全国文化产业工作先进集体”称号。卫生服务能力增强，乡村、社区公共卫生服务体系进一步健全。覆盖城乡的食品药品综合监管网络基本形成，问题奶粉事件应急处置及时到位。竞技体育实现历史性突破，我市运动员吴静钰、金紫薇在北京奥运会上勇夺金牌，为瓷都争得了荣誉。老年体育活动中心投入使用。第七届村（居）委会换届选举工作全面完成。积极开展向四川地震灾区捐助和支援工作。国防后备力量进一步加强，民兵预备役部队积极参建。同时，计划生育、人事编制、监察、供销、水文、气象、民族宗教、新闻出版、双拥、老龄、妇女儿童、残联、人防、档案、地方志、金融、保险、烟草等各项事业也都取得了新的进步。

行政效能不断提高。主动接受人大及其常委会的法律监督、政协及其常委会的民主监督。及时办理人大代表意见建议89件，政协提案107件。加强规范性文件审查，完成政府规范性文件、其他文件和协议的法律审查195件。统计调查工作得到明显加强，整体功能进一步提升。审计监督机制逐步健全，职能进一步完善。政府行政效能不断提高，经济发展环境不断优化，注册办证“一站式”审批服务水平不断提升，外商投诉问题集中处理协调机制不断健全。国务院批准授予我市正处级以下人员因公出入境审批权。“市长短信”平台建设不断完善。六项考核成效显著，在全省排位前移。政风行风建设扎实推进，在全省民主评议政风行风活动中，我市总成绩名列全省前茅，19个部门进入全省本系统前三名，其中市经贸委、市公安局、市建设局、市广电局、市体育局和市环保局获第一名。

各位代表，过去的一年，我市经济社会发展所取得的成绩来之不易。这是省委、省政府和市委坚强领导的结果，是市人大、市政协大力支持、有力监督的结果，是全市人民团结拼搏、辛勤劳动的结果，也是与历届政府打下的扎实基础分不开的。在此，我代表市人民政府，向全市广大工人、农民、知识分子、干部和离退休老同志，向各民主党派、工商联、无党派人士和社会团体，向驻市解放军、武警官兵和公安干警，向所有关心、支持瓷都发展的海内外朋友，表示衷心的感谢，并致以崇高的敬意！

在看到成绩的同时，我们也清醒地看到经济社会发展中还存在不少困难与问题。主要是：经济总量仍然偏小，总体发展水平还不高；项目库建设还不够完善，项目后劲有待增强；就业压力进一步加大，群众持续增收比较困难；经济发展环境和行政效能仍有待进一步改善和提高等。所有这些，我们将采取更加有力的措施，努力加以克服和解决。

二、2009年的工作任务和主要措施

今年是建国60周年，也是推进“十一五”规划顺利实施的关键一年，保持和实现全市经济社会平稳持续较快发展是我市今年工作的关键和首要任务。

2009年市政府工作的总体要求是：坚持以科学发展观为指导，积极落实中央扩大内需、促进经济增长的宏观调控政策，把保增长、扩内需、调结构更好地结合起来，以工业化为核心，以大开放为主战略，以项目建设为重点，继续增加投入、做大总量，以大项目推动大发展，以高投入拉动高增长，以促改革创新增活力，以重民生实现大稳定，努力实现全市经济社会平稳持续较快发展。

2009年全市国民经济和社会发展的主要目标是：全市生产总值增长13%；财政总收入增长15%；全社会固定资产投资增长40%；规模以上工业增加值增长20%；社会消费品零售总额增长16%；出口总额增长20%；实际利用外资增长30%；实际利用内资增长40%；城镇居民人均可支配收入增长10%；农民人均纯收入增长8%；城镇登记失业率控制在4.5%以内；单位GDP能耗下降5%；居民消费品价格指数控制在103.9%以内；人口自然增长率控制在8‰以内。

实现上述目标，要认真学习贯彻好胡锦涛总书记在我省考察期间的重要讲话精神，保增长、保民生、保稳定，弘扬井冈山精神。要把固定资产投资增长放在今年工作的第一位，以产业项目建设为重点，保持增速领先，做大总量进位，坚定信心，团结拼搏，以快制胜，乘势而上，力争各项工作进入全省中上水平。

（一）增加投入做大总量，全力推进项目建设

狠抓项目、增加投入，既是积极把握中央宏观调控政策，贯彻落实省委省政府的决策部署，也是立足我市发展实际，利在当代、功在长远的重大战略举措。我们既要做顶天立地的大项目，种好“大树”，又要做铺天盖地的中小项目，培育“灌木、草丛”，全年完成城镇以上固定资产投资300亿元以上。

推进产业项目建设。重点培植做大光伏产业、航空汽车及机电产业、陶瓷产业、医药化工产业、电子和家电产业等，积极推进已开工的多晶硅、长虹家电、汽车零配件、LED照明、金龙化工、莱特、欧文莱等项目建设进度，争取早日投产。加快推进已投产的乐华、特地、金意陶等项目的扩产建设。抓好已签约的投资10亿元的远红外陶瓷、7亿元的景德镇信息科技产业园、投资1亿美元的万裕电子、投资5亿元的宁波吉品电子等项目的开工、建设。积极争取投资10亿元的锂电子新能源及其适配器产业项目和投资1亿元的LED孵化中心等项目的签约、落地、开工。

推进基础设施项目建设。加快交通基础设施项目建设，开工建设九景衢铁路和皖赣复线改造工程、德昌高速；续建南环高速；建设完工湘官一级公路、景瑶公路、吴乐秧公路，完成农村公路建设160公里，实施县至乡油路改造110公里，加快推进农村区乡站、村庄候车亭项目建设；积极推进机场扩建及附属工程建设，完善景德镇支线航空基地功能。加快能源项目建设，开工建设电厂“上大压小”项目；推进天然气管网建设，争取年底通气；完善电网建设，加快500千伏景德镇变电站和光伏变电站建设进度，确保110千伏马鞍山变电站上半年投入运营，220千伏南山、110千伏宝石、梧桐变电站年内竣工投入运营，加快220千伏涌山和110千伏景东变电站开工建设。加快农林水利基础设施建设，开工建设浯溪口水利枢纽工程、昌江和乐安江治理工程、玉田水库扩建工程，继续推进城市防洪、病险水库除险加固、灌区配套改造等工程。加快城市基础设施项目建设，加快推进棚户区改造、第四水厂二期扩建、市第二垃圾处理场、供水管网改造二期工程、老城区排水主干管道改造等项目。

推进社会事业项目建设。推进科技创新项目，争取景德镇陶瓷工业园区列入国家新材料科技创新基地；推进民用直升机产业化科研攻关工程、陶瓷窑炉节能技术改造、特种陶瓷材料研究及应用开发、青花玲珑等传统特色陶瓷产业技术创新等项目。推进教育类项目建设，完善城乡教育布局。建成新五中、梨树园小学并投入使用；加快推进一批中小学校整合扩建；加大农村初中改造力度，实施市职业教育示范校、县级职教中心和特殊教育学校新扩建工程。推进文化、卫生、体育、计生等项目建设，加快市妇幼保健院、市中医院、市第五医院建设；积极争取国家农村卫生服务能力建设项目、农村广播电视无线覆盖工程和数字电视无线覆盖体系工程等项目；完成光明电影院、体育中心建设。加快建设全国节水型城市。积极申报资源枯竭型城市试点，努力向循环经济试点城市迈进，全力推进省级综合配套改革自主创新试点城市申报工作，争取国家和省政策支持。

（二）坚持大开放主战略，全面提升开放型经济水平

继续坚持以大开放促进大发展，进一步拓展对外开放的广度和深度，提升开放型经济水平。

加快园区平台建设。围绕在全省园区争先进位的目标，突出园区特色，构建产业配套体系，以园区主导产业择商选资，提高产业聚集度和园区投入产出比。市高新区和乐平工业园要力争进入全省一类园区行列。陶瓷工业园固定资产投资、规模以上工业增加值、财政总收入要保持高速增长势头，力争进入全省二类园区。

全力推进招商引资。发挥各方主观能动性开展招商引资，更广泛地吸纳社会资本。树立“招商就是找项目”的发展理念，把引进工业项目作为招商重点推进，把引进外资作为难点突破，围绕我市产业招商规划重点引进一批配套项目、上下游项目，扩大产业配套，形成企业集群。同时，抢抓国家调整加工贸易政策机遇，做好承接转移的各项工作。改进对外贸易增长方式，不断扩大对外贸易，培育外贸出口企业和人才队伍。

提高招商项目成效。做好招商项目衔接跟进的各项服务，落实追踪服务的问效机制，不断优化开放型经济发展环境。在抓好项目的签约、落地建设的同时，高度重视项目的开工、投产，确保招商项目成效。加强签约落户项目调度，对每一个已落户的项目查实效，对正在落实的项目查进度，对没有落实的项目查原因。及时了解企业所需，解决项目在招商、建设过程中的各种困难和问题，确保落户项目早开工、早投产、早见效。

（三）以农民增收为目标，努力推进农村改革和发展

把“三农”工作摆在更加突出的位置，围绕稳粮、增收、强基础、重民生，全面推进“三农”工作。力争今年农业总产值增长6%，粮食稳产在50万吨以上，力争55万吨。

稳定粮食生产。加大以农田水利基本建设为重点的农业基础设施建设，深入实施“百万亩农田增灌”工程，抓好优质粮食产业工程建设，落实病险水库除险加固资金投入，搞好灌区渠系配套，推进农田灌溉工程建设。认真组织好2009年度国家农业综合开发土地治理项目及产业化经营项目建设。坚持最严格的耕地保护制度，确保耕地“占补平衡”。继续实施“造地增粮富民”工程，提高粮食综合生产能力，在稳定粮食生产的同时，抓好优质粮食生产基地建设，优化粮食生产结构，提高优质稻比重，确保粮食播种面积稳定在137万亩。

促进农民增收。认真落实各项强农惠农政策，提高农资综合直补、良种补贴、农机具补贴等标准，完善与农资价格上涨挂钩的农资综合补贴动态调整机制，落实好提高粮食最低收购价格政策，充分发挥收购价格对增产增收的促进作用。大力发展现代农业，抓好乐平蔬菜、浮梁茶叶、昌江养殖三大特色产业建设。重点扶持养殖业，以创办万头养猪场、百头养牛场等发展养殖大户，建设畜牧小区，创建养殖专业村，加快畜牧业发展。继续抓好农业产业化“121”工程和“双十双百双千”工程，重点抓好省级以上龙头企业，促进龙头企业扩张，带动农民增收。扎实做好农民就业、创业工作，安排专项资金免费培训农民工，增强返乡农民工就业本领，并从税收优惠、用地审批、项目扶持等方面予以支持，鼓励和引导农民自主创办各种经济实体，实现由劳务向创业的转型。

扎实推进新农村建设。围绕新农村建设“二十字”方针，坚持“政府主导、农民主体、干部服务、社会参与”的工作机制和“市级领导挂帅、专门人员指导、具体部门帮扶、社会各界支持”的模式，充分发挥农村基层组织在新农村建设中的作用，继续加大对新农村建设试点工作的扶持力度，做到建一个成一个，提高档次，巩固成果，抓点连线成片。坚持把发展经济、富裕农民作为新农村建设的根本任务，加大“一村一品”和产业化建设力度。实施以庭院绿化、道路绿化、村旁绿化、村庄垃圾无害化处理为主要内容的村庄清洁绿化工程，不断改善农民居住和生活条件。

增强县域经济发展活力。进一步把县域经济摆到全市经济社会发展大局中谋划，加强支持服务，进一步激发县域经济发展活力。依托资源优势和地域特色，因地制宜确定发展定位和方向，加快产业聚集和区域特色经济发展，打造特色产业体系。乐平市要依托现有工业基础，在加强环境保护的前提下，着力发展化工、医药、机械、矿产和建筑陶瓷等产业，力争工业总量在全省争先进位。浮梁县要切实加强陶瓷工业园区和三龙工业基地建设，以发展高档精品陶瓷为重点，做大陶瓷产业规模；以申报国家级生态县为契机，着力做好生态旅游和茶文章。昌江区要紧扣我市“城市西扩、工业南聚”的战略机遇，积极参与城市化和大中型企业的协作分工，配套城市生产、生活。珠山区应加大社区建设力度，主攻三产，服务城市。推进全市城镇化进程，加快涌山、众埠、竟成、吕蒙、洪源、湘湖等一批中心镇建设，提升城镇的综合功能，增强聚集效应。

（四）提升工业发展水平，加快推进工业化核心战略

大力实施工业强市战略，促进产业经济集约发展，不断做大工业总量，努力实现工业发展新突破。2009年，全市规模以上工业增加值占GDP比重力争提高3个百分点。

做大做强优势企业。选择一批有市场、有订单、有信誉、有效益的企业进行重点扶持，落实好相关配套支持政策。对缺乏流动资金的，提供中小企业信用担保融资支持，破解企业发展难题，争取培植一批产值过亿企业，今年全市力争新增规模以上工业企业120户。

提高企业“四率”水平。大力开展以节能减排增效、节支降本增产为主要内容的“双增双节”活动，引导企业不断提高劳动生产率、人均创利率、资金利润率、资源能源利用率，苦练内功，不断提高企业赢利水平，增强企业综合竞争力，全市工业经济效益综合指数达到220%。

做大陶瓷产业总量。以陶瓷工业园区为依托，突出项目建设，做大陶瓷产业总量。2009年全市陶瓷工业总产值突破100亿元，陶瓷出口1亿美元，陶瓷税收超亿元。提升国家日用及建筑陶瓷工程技术研究中心功能，加快陶瓷科技创新，促进陶瓷科技成果转化。全力办好2009景德镇国际陶瓷博览会，努力构建大陶瓷市场体系。加大陶瓷市场整顿，提升陶瓷设计水平，推进陶瓷品牌战略。

（五）加快改革创新，增强经济发展活力

坚持以改革破解难题、转变机制、激发活力、提升水平，着力构建充满活力、富有效率、有利于科学发展的体制机制，勇于攻坚破难，务求在重点领域和关键环节取得实质性进展。

稳步推进农村各项改革。抓好土地承包经营权证换发补发，实现承包合同、经营权证“两个100%”落实到户。坚持依法自愿有偿规范的原则，发展适度规模经营，支持专业大户、农民专业合作社等规模经营主体发展。抓好林权制度配套改革，促进林业经营权规范有序流转。强化乡镇政府社会管理和公共服务职能，加快县乡财政管理体制改革，推行“乡财县管”，积极稳妥化解乡村债务，力争到年底基本化解全市农村义务教育债务。

全面推进国有企业改革。坚持“因企制宜、一厂一策”，力争今年基本完成国企改革任务。重点做好省属下放12户企业的改制，推进14户企业政策性破产工作。积极寻找战略投资者，重点推进陶瓷股份公司、德宇集团、九州洁具厂、东风药业、江维高科等重点企业招商引资和企业改制。同时，继续做好资产包回购工作，加大债权清收力度。

积极推进财政改革。深化国库集中支付制度改革，确保81个移交单位的财务工作平稳过渡。进一步完善非税收入管理，积极推进执收执罚部门收支脱钩改革。启动预算单位公务卡改革。推进财政票据电子化改革，力争上半年基本完成改革任务。

深化投融资体制改革。加快扶持重点企业上市步伐，激活资本市场。加快组建城市商业银行，积极引进外来商业银行。组建景德镇投资公司。完善县（市、区）、园区投融资平台建设。做好机关事业单位经营性资产统一经营管理工作，增强政府投融资能力。积极寻找符合信贷政策的项目，争取国债转贷资金。设立产业投资引导基金，引导社会资本投资我市符合产业发展方向、处于种子期和起步期的企业，解决企业扩张之前的资金瓶颈问题。加强政银企协作，鼓励金融机构扩大信贷投放规模。

（六）突出陶瓷文化特色，大力发展文化旅游

充分发掘陶瓷历史文化资源，打好文化牌，做足瓷文章，促进陶瓷文化资源优势向旅游产业优势转化，全面提升瓷都文化旅游的整体水平，力争全市接待国内外游客突破1000万人次，旅游总收入突破50亿元。

加快景区景点建设。进一步完善陶瓷历史文化博览区、御窑厂、高岭-瑶里风景区、浮梁古县衙、洪岩风景区等重点景区景点功能建设，打造景德镇王牌景区。挖掘、利用老城区陶瓷历史文化资源，突出抓好民窑博物馆、祥集弄民居和三闾庙古码头等老城区旅游项目建设；加快建设莲社路陶瓷精品一条街；推出若干条以老瓷行、老坯房、老里弄、古民居等为特色的“老景德镇”精品旅游线路。积极做好陶瓷文化旅游集聚区旅游基础设施建设、陶瓷旅游和历史文化保护、旅游商品研发集散中心、游客服务中心、红色旅游等项目包装、申报和争取工作，加快中国瓷园旅游景区配套项目的招商引资工作。

发展文化创意产业。启动文化创意产业园区的保护性规划，对具有历史性保护价值的近现代遗存瓷厂成片厂房区域进行保护和开发。出台扶持文化创意产业发展政策，精心打造一批以陶瓷科研院、所、校和企业为重点的景德镇陶瓷文化创意园区。加强非物质文化遗产保护，继续做好传统制瓷代表性传承人调查、宣传、管理工作，加强传统陶瓷技艺的保护和传承，积极开展陶瓷工业文化遗产和有代表性建筑物的普查和保护工作。

加强旅游产品开发。积极策应国家旅游局推行的“国民休闲计划”和“生态游”活动，推出丰富多彩的旅游节庆活动和大型特色演出活动，丰富旅游纪念品市场，打造陶瓷文化旅游产品和休闲度假旅游产品。大力发展陶瓷工业旅游、陶瓷文化体验游、文化休闲旅游、乡村农家乐旅游等特色旅游项目，形成多样化的旅游产品体系，通过旅游产品开发提升旅游产业竞争力。

加大旅游宣传推介力度。坚持政府形象宣传与企业产品促销相结合，品牌营销与线路推荐并重，国内和国际两大市场共拓，整体包装、组合营销、重点推介。加强与主流媒体的合作，通过新闻媒体宣传、举办推介活动、旅行社采风等多种渠道和形式，提高瓷都知名度，扩大瓷都旅游影响力。更多地组织邀请新闻媒体、知名人士来景德镇写作、拍摄和进行影视创作。积极举办旅游推介会，支持和引导旅游企业参加各类旅游交易会，推进区域旅游合作，形成开放、联动的旅游客源网络。

（七）策应鄱阳湖生态经济区建设，全面推进山水生态文化名城建设

按照“城市向西扩展、工业向南聚集”的发展格局，坚持千年古韵与现代气息相结合，打造城在山中、水在城中、人在画中的山水、生态、文化特色宜居城市。

科学规划城市。继续以创建“国家五城”为目标，以完善山水、生态、文化特色和基础设施、功能设施为重点，完成《景德镇市城市总体规划（2008—2030年）》、《景德镇市土地利用总体规划》、《景德镇市近期建设规划》和《景德镇市城镇体系规划》等重大规划和专项规划编制，形成全覆盖的规划控制网络，并严格执行规划。完成城市规划展示馆布展。启动城市地下管网普查，实施地下管网等基础设施建设的规划管理。注重城市设计，规划建设一批体现历史文化特色和城市风貌的建筑小品和城市景观。

重点推进城市建设。重点打造行政中心；加快启动沿江西岸片区景观带项目建设；加快推进景德大道、景东大道全线贯通工程；加快以昌南大道为轴心、沿生态路和206国道扩展的新城区建设；加快推进800亩城市森林公园建设。完成新平阁、昌南阁、景德阁建设；扎实推进生态公园、松涛公园、广场商贸大厦、新天地广场、人民剧场、公安指挥中心、“一江两岸”和广场立面改造等一批重点工程建设。稳步开展25万平方米老城区危旧住房和厂矿棚户区改造。改善城市道路交通条件，完成解放路、银曙路、太平巷路、中山北路延伸、麻石弄等5条主干道和下窑路、昌虹路、凤凰山路、茶叶坞、窑建厂路等5条次干道改造。完善市政设施，建成东市区消防站，改造市区里弄路灯，新建20座公厕和5个垃圾中转站，免费开放城区公厕。

加强城市经营管理。积极争取用地指标，保障重点项目、工业园区建设用地。做好土地储备、出让和融资工作，加大土地回购工作力度，提高土地市场运作效益。今年计划出让经营性土地1500亩，实现净收益5亿元。积极创建国家卫生城市、江西省第三届文明城市。进一步创新城市管理机制，逐步建立科学、系统、完整的城市管理体系。继续推进无证私房清理整顿，严厉打击违法用地和违章建筑，实现城市建设有序发展。实施标准化农贸市场示范工程，改造一批农贸市场。大力发展矿业经济，重点做好陶瓷原料的勘探和锰矿等矿产资源的开发和经营工作。

（八）坚持以人为本，扎实推进民生工程

本着“量力而行、尽力而为、统筹兼顾、创造条件、积极有序、办好实事”的原则，坚持新增财力“四个倾斜”，突出“雪中送炭”，努力解决与群众生产生活密切相关、直接涉及群众切身利益的问题。全市共安排民生工程资金10亿元，重点做好八个方面工作。

一是扩大就业、促进创业。实施积极的就业政策，完善就业服务体系，千方百计增加就业，鼓励创业。全市城镇新增就业2.16万人；积极帮助困难群众就业，“4050”人员新增就业1500人，零就业家庭就业安置率100%；新增转移农村劳动力2.1万人；城镇职业培训1.7万人，其中创业培训2000人；城乡残疾人培训610人；发放小额贷款1.11亿元,其中支持个人创业6800万元。

二是健全社会保险制度。努力扩大社会保险覆盖面，加强社保基金征缴。积极做好城镇职工参加基本养老保险工作，基金征缴总额4.83亿元；推进被征地农民养老保险试点，参保人数2万人；扩大失业保险覆盖面，参保人数13.96万人，基金征缴总额2654万元。

三是提高社会救助水平。努力扩大救助范围，健全救助体系，提高救助标准。城市和农村低保补差分别达到每人每月155元和65元；完善城乡临时救助制度；提高精简退职老职工救济金水平，其中城市达到200元、农村达到160元；城镇“三无人员”供养标准达到每人每月300元；积极落实地方救灾配套资金。

四是加大医疗保障力度。积极完善医疗、工伤保险和医疗救助制度，努力提高公共卫生服务水平。全市城镇职工基本医疗保险参保31.3万人；城镇职工工伤保险参保20万人，农民工参加工伤保险5.03万人；城镇居民基本医疗保险覆盖率达到100%；完善新型农村合作医疗制度，统筹基金使用率85%以上；免费提供社区公共卫生服务卡420万元。

五是关心困难群体就学就医。义务教育阶段免学杂费和免费提供教科书学生数均达到19万人；资助普通高中家庭经济困难学生2920人；资助中等职业教育家庭经济困难学生8300人；资助考入大学家庭经济困难学生797人；免费入读技工学校家庭经济困难学生180人；建设乡镇文化站8个；村卫生室（所）达标95个、乡镇中心卫生院达标2个、县级综合医院达标1个；社区卫生服务机构达标29个。

六是加强保障性住房建设。进一步推进经济适用住房和廉租住房建设，并为农村困难群众提供住房帮助。建设经济适用住房1140套，新建廉租住房1250套，享受廉租住房租赁补贴8644人；为农村困难群众新建、重建住房176户。

七是推进扶贫开发。移民扶贫搬迁246人，整村推进重点扶贫村12个。

八是提高农村群众生产生活水平。解决23万农村人口饮水安全问题；农村使用沼气新增4000户；积极开展送戏下乡、为农村放映电影、组织农村自办文体活动；建设农民体育健身场所14个、农家书屋54个，农村家庭生活垃圾处理场所190个，开工建设渡改桥项目17个。

（九）统筹经济社会，推进各项事业协调发展

统筹兼顾，合理安排，调动公共资金，引导社会各方力量，支持重点领域，加强薄弱环节，积极发展各项社会事业，努力形成社会和谐人人有责、和谐社会人人共享的生动局面。

统筹推进社会事业。不断提高科技创新能力，加强与中科院等大型科研院所合作。加强科技项目申报工作，重点申报国家中小企业创新基金、国家重点新产品和火炬计划项目。优先发展教育事业，巩固提高九年制义务教育，加强农村义务教育控辍保学，初中辍学率控制在2.4%以下，高中毛入学率70%以上，新增7所高中进入省重点或省重点建设中学行列。积极推进景德镇陶瓷学院申报博士点、景德镇高专升入本科院校，协调发展职业教育、特殊教育、学前教育和老年教育。繁荣文化事业，在北京举办建国60周年景德镇陶瓷发展成就展。组织创编一批优秀作品和文艺精品，繁荣文艺创作和演出，开展形式多样的社会文化活动。完善文化遗产保护机制，开展第三次文物普查，提高文物展示、交流和利用效率。提高公共卫生服务水平，加强疾控机构能力建设，加强重大疾病预防控制，完成市传染病医院建设任务。加强医院管理，提高医疗服务质量和技术水平，改进医德医风，积极争创“三甲”医院。大力推进国防后备力量建设。争创“全国双拥模范城”五连冠。积极开展群众性体育活动，发展竞技体育，提高人民身体素质和体育竞技水平。加强档案依法管理。深入开展食品药品安全专项整治。继续稳定低生育水平，不断提高人口素质，综合治理出生人口性别比偏高问题。

加强生态环境保护。扎实做好节能减排、资源节约和环保工作。化学需氧量排放量削减1300吨，控制在2.22万吨以内；二氧化硫排放量削减2000吨，控制在4.59万吨以内。建成市第二污水处理厂和乐平、浮梁污水处理厂，加快乐平、浮梁垃圾处理场建设。加大节能减排和窑炉改造力度，实施工业污染治理工程，深入开展整治违法排污企业环保专项行动。大力实施造林绿化“一大四小”工程，围绕到2010年全市森林覆盖率达到65%的目标，今年完成造林绿化15.32万亩。推进生态功能保护区创建工作，重点抓好景德镇中国檵树园、德宇龙山园、植物园等项目建设，推进现有自然保护区升级，积极创建国家和省级生态保护区。

推进和谐平安瓷都建设。严厉打击严重刑事犯罪活动，增强群众安全感。完善社会治安防控体系建设，有效预防和减少各类违法犯罪行为。切实加强和改进信访工作，坚持源头治理，畅通信访渠道，规范信访行为，完善工作制度，切实把信访问题解决在基层，把各种矛盾化解在萌芽状态。关心困难群众，搞好法律援助。健全突发事件应急管理机制，完善各种应急预案，提高政府应对复杂局面和处置突发公共事件能力。健全安全生产工作责任制，夯实安全生产基础，杜绝重、特大安全生产事故发生。

（十）以行政效能建设为重点，加强政府自身建设

开展深入学习实践科学发展观活动，按照科学执政、民主执政、依法执政的要求，以依法高效、创建最优发展环境为目标，以提高项目审批和建设效率为重点，加强政府自身建设，打造“和谐、创新、务实、高效、自律”政府。

紧抓第一要务，增强发展能力。牢固树立“工作以经济建设为中心，经济建设以项目为核心，不抓项目就是失职，不会抓项目就是不称职”的理念，增强发展的预见性、前瞻性、主动性，善于在危机中寻找机遇，在机遇中寻找项目。进一步提高抓项目、谋项目、上项目的工作水平，把握国家政策导向，熟悉和规范项目论证、立项、申报、实施等程序，增强资本运作本领，用抓项目的本事来真正体现抓经济工作的能力。

提高行政效能，优化政务环境。深入开展机关“效能年”建设活动，建立健全行为规范、运转协调、公正透明、廉洁高效的机关运行机制，确保政令畅通，打造良好的政务环境。加强和完善行政服务中心建设，推行部门的行政审批职能向一个科室集中、部门的行政审批科室向行政服务中心集中。加快项目审批流程，建立重大项目联审机制。继续推进电子政务建设，畅通“12345”为民服务热线。牢固树立“支持企业有责，成就企业发展有功”的理念，最大限度地减少检查、评比和会务，把更多的时间和精力用在为企业服务上，真心实意为企业着想，千方百计帮助困难行业和困难企业渡过难关，满腔热情为企业发展鼓劲、撑腰、壮胆，形成浓厚的“亲商、安商、富商”氛围。

坚持依法行政，打造诚信政府。进一步完善重大决策的规则和程序，推进依法决策、科学决策和民主决策。自觉接受人大及其常委会的法律监督和政协的民主监督，认真听取各民主党派、工商联、无党派人士和人民团体的意见，接受新闻舆论和社会公众监督。强化行政执法监督，严格实行执法责任制和执法过错追究制。重信用、守合同，以改善环境的实际成效取信投资者，以明晰的责任体系塑造政府诚信形象。推行政务公开，规范公共资源交易行为，进一步完善工程招标、土地招拍挂公示制度，坚持民生工程资金阳光操作，使社会普遍关注的热点问题始终处于人民群众的监督之下。

加强自身建设，严格廉洁自律。认真落实党风廉政建设责任制，深入开展反腐倡廉。加强政府班子建设，落实干部廉洁自律各项规定。加强投资项目审计监督，着力推进重点领域和关键环节反腐败预防工作。大力发扬艰苦奋斗、勤俭节约作风，坚决制止奢靡之风和铺张浪费行为。严格控制出国出境考察活动，严禁用公款相互走访、相互送礼、相互宴请。加强公务员教育和管理，廉洁从政，勤政为民。

各位代表，2009年是共和国成立60周年，做好今年的各项工作，意义重大，使命光荣！让我们高举中国特色社会主义伟大旗帜，更加紧密地团结在以胡锦涛同志为总书记的党中央周围，全面贯彻落实科学发展观，在省委、省政府和市委的领导下，进一步解放思想，坚定信心，团结拼搏，排难前进，为保持全市经济社会平稳持续较快发展，实现瓷都新跨越作出更大的贡献！
